# Anti-Inflammatory Effects and Photo- and Neuro-Protective Properties of BIO203, a New Amide Conjugate of Norbixin, in Development for the Treatment of Age-Related Macular Degeneration (AMD)

**DOI:** 10.3390/ijms24065296

**Published:** 2023-03-10

**Authors:** Valérie Fontaine, Christine Balducci, Laurence Dinan, Elodie Monteiro, Thinhinane Boumedine, Mylène Fournié, Vincent Nguyen, Louis Guibout, Justine Clatot, Mathilde Latil, Stanislas Veillet, José-Alain Sahel, René Lafont, Pierre J. Dilda, Serge Camelo

**Affiliations:** 1 Sorbonne Université, INSERM, CNRS, Institut de la Vision, 17 Rue Moreau, 75012 Paris, France; valerie.fontaine@inserm.fr (V.F.);; 2 Biophytis, Sorbonne Université, BC9, 4 Place Jussieu, 75005 Paris, Franceserge.camelo@biophytis.com (S.C.); 3 Fondation Ophtalmologique Rothschild, 29 rue Manin, 75019 Paris, France; 4 Department of Ophthalmology, School of Medicine, The University of Pittsburgh, Pittsburgh, PA 15213, USA

**Keywords:** A2E, AMD, eye, norbixin-conjugate, pharmacokinetics, PPARs, RPE

## Abstract

9′-*cis*-norbixin (norbixin/BIO201) protects RPE cells against phototoxicity induced by blue light and *N*-retinylidene-*N*-retinylethanolamine (A2E) in vitro and preserves visual functions in animal models of age-related macular degeneration (AMD) in vivo. The purpose of this study was to examine the mode of action and the in vitro and in vivo effects of BIO203, a novel norbixin amide conjugate. Compared to norbixin, BIO203 displays improved stability at all temperatures tested for up to 18 months. In vitro, BIO203 and norbixin share a similar mode of action involving the inhibition of PPARs, NF-κB, and AP-1 transactivations. The two compounds also reduce IL-6, IL-8, and VEGF expression induced by A2E. In vivo, ocular maximal concentration and BIO203 plasma exposure are increased compared to those of norbixin. Moreover, BIO203 administered systemically protects visual functions and retinal structure in albino rats subjected to blue-light illumination and in the retinal degeneration model of *Abca4^−/−^ Rdh8^−/−^* double knock-out mice following 6 months of oral complementation. In conclusion, we report here that BIO203 and norbixin share similar modes of action and protective effects in vitro and in vivo. BIO203, with its improved pharmacokinetic and stability properties, could be developed for the treatment of retinal degenerative diseases such as AMD.

## 1. Introduction

Age-related macular degeneration (AMD) is the commonest cause of severe visual loss and blindness in developed countries among individuals aged 60 and older and remains a major medical need [1,2]. AMD slowly progresses from early AMD to intermediate AMD (iAMD), which further evolves to late-stage neovascular AMD and/or geographic atrophy (GA) [2]. It has been known for many years that carotenoids, such as lutein and zeaxanthin, are constitutive of the macula, and dietary apocarotenoids such as crocetin, and 9′-*cis*-norbixin (norbixin, pharmaceutical ingredient of BIO201) preserve the retinal architecture and visual functions [2].

Norbixin is a 6,6′-di-*apo*-carotenoid extracted from annatto (*Bixa orellana*) seeds [3]. We have previously demonstrated that norbixin protects porcine primary retinal pigmented epithelial (RPE) cells from phototoxicity induced by blue-light illumination coupled with A2E exposure in vitro [4]. Norbixin modulates inflammation, as demonstrated by its inhibitory effect on A2E-induced expression of IL-6 and IL-8 and by its effect on the transactivation of NF-κB and AP-1. In addition, our previous observations demonstrated that norbixin impairs VEGF expression induced by A2E [5]. Norbixin also reduces the accumulation of A2E by primary porcine RPE cells in vitro [4]. In vivo, norbixin possesses anti-inflammatory and antioxidant properties [6]. In agreement with the reported role of inflammation during retinal degeneration, we have shown that norbixin is beneficial in various in vivo animal models of AMD [4,7]. Indeed, norbixin administered systemically is neuroprotective against blue-light-induced retinal degeneration in rats and BALB/c mice [4,7]. Moreover, 5- to 6-months oral treatment by complementation of *Abca4^−/−^ Rdh8^−/−^* double-knockout mice with norbixin-containing chow results in neuroprotection and partially preserves the function of both rods and cones in vivo. Oral complementation with norbixin also reduces A2E and lipofuscin accumulation in RPE cells in these animals [7]. The beneficial effects of norbixin are potentially associated with its interaction with several nuclear receptors: PPAR-γ, PPAR-α, PPAR-β/δ, and also RXR-α [5,8]. We proposed that norbixin behaves as a neutral antagonist of these nuclear receptors, thus inhibiting their transactivation induced by A2E [5]. Based on all these properties, norbixin or related carotenoids appear promising to treat the intermediate form of AMD, for which an effective drug is still lacking [2].

Despite all these interesting properties, we observed that norbixin stability at all temperatures tested and its ocular bioavailability were not acceptable for further industrial/clinical development (unpublished data). Therefore, we aimed to identify possible hemisynthetic compounds derived from norbixin retaining its biological effects on RPE cells and in vivo but with improved stability and bioavailability. We produced a set of norbixin amide conjugates by hemisynthesis from bixin. We prepared a range of primary or secondary amides, which were assayed by the same battery of tests: in vitro bioactivity, pharmacokinetic parameters in plasma, and ocular exposure, as well as stability. We found that most of the resulting compounds had a better pharmacokinetic profile and an improved tropism for the eye when compared with norbixin (patent Dinan et al., 2019; US 17/788,534). In addition, some of these compounds showed a retinal pigment epithelial (RPE) photo-protective activity equivalent to or greater than that of norbixin. In the present study, we describe the effects of one of these conjugates (BIO203) in in vitro and in vivo models of AMD. BIO203 stability and ocular pharmacokinetics are presented here in comparison with those of the parent compound, norbixin (BIO201).

## 2. Results

### 2.1. BIO203 Is More Stable Than Norbixin (BIO201)

**Figure 1:**
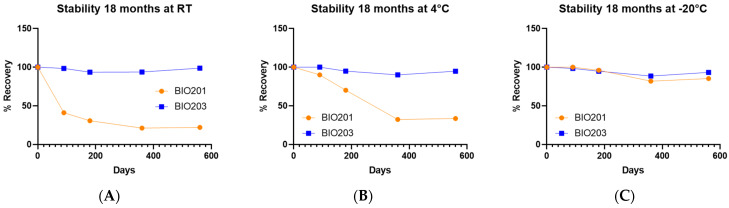
Compared stability of powders of BIO203 and norbixin (BIO201) at RT, 4 °C, and −20 °C for a period of 18 months. (**A**) Percent of recovery of BIO203 (in blue) and BIO201 (norbixin in orange) at room temperature (RT). (**B**) Percent of recovery of BIO203 and BIO201 at 4 °C and (**C**) at −20 °C.

We evaluated the stability of BIO203 compared to norbixin at room temperature (RT) (Figure 1A), +4 °C (Figure 1B), and −20 °C (Figure 1C) for a period of up to 18 months. After 3 months at RT, we already observed a very significant decrease in norbixin stability, with only 41% left of the initial quantity. The amount of norbixin kept decreasing at 6- and 12-month time points but stabilized between 12 and 18 months to 22.1% of the initial quantity of norbixin (Figure 1A). Setting the temperature at 4 °C slightly improved the stability of norbixin. Nevertheless, at 3 months, there was already a loss of 10% of the compound. This loss was still ongoing at 6 months (−20.8%) and 12 months (−66.4%) (Figure 1B). As for the stability at RT, no further decay was observed between 12 and 18 months at +4 °C (Figure 1B). At −20 °C, norbixin appeared stable for up to 6 months; however, there was a significant loss of compound after one year (−18.1%) that did not worsen during the last 6-month period (between 12 and 18 months; Figure 1C). Therefore, we observed a natural loss of norbixin at all temperatures, but this was limited and delayed when negative temperatures were applied. By contrast, BIO203 was stable at all temperatures tested for at least 18 months (Figure 1A–C).

#### 2.1.1. In Vitro, BIO203 and Norbixin Are Equally Effective in Protecting RPE Cells against Phototoxicity Induced by Blue-Light Exposure in the Presence of A2E

**Figure 2:**
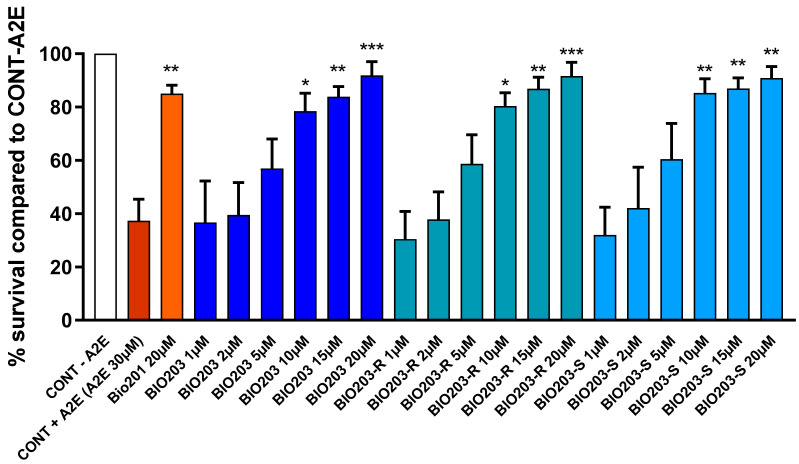
Dose-response of the photoprotection induced by BIO201, BIO203 (racemate), and its enantiomers R and S on RPE cells cultivated in the presence of A2E and blue-light exposure. A2E was used at 30 μM and BIO203 and its enantiomers at the concentrations indicated. Bars represent mean ± s.e.m. with n = 3 per group. * *p* < 0.05, ** *p* < 0.01, *** *p* < 0.001, compared to CONT + A2E (One-way ANOVA, Dunnett’s post-test).

We demonstrated previously that norbixin inhibits the phototoxicity of primary RPE cells induced by A2E in the presence of blue light [4]. Here, we confirmed our previously published results [4], showing that norbixin treatment at 20 μM promotes the survival of more than 80% of RPE cells subjected to blue-light illumination in combination with A2E exposure (Figure 2). In addition, we show here that increasing doses of BIO203 also protect RPE cells illuminated in the presence of A2E (Figure 2). BIO203 protective effect was observed from 10 μM and increased at 15 and 20 μM. Interestingly, BIO203 reaches its maximum efficacy at 15 μM while the highest protection by norbixin is obtained at 20 μM. Since BIO203 contain an asymmetric carbon in the amide radical, BIO203 is a racemic compound composed of two enantiomers called BIO203-R and BIO203-S obtained by the addition of the 3-MP-R and 3-MP-S radicals, respectively. We tested whether the protective activity of BIO203 lay in one of these enantiomers or if it was shared by both. We observed that both enantiomers have the same protective activity as BIO203 at all concentrations from 10 to 20 μM (Figure 2). This indicates that there is no difference between enantiomers and BIO203 (racemic mix) activities, and it suggests that the effect of the racemic compound (BIO203) should be equally shared by both of its enantiomers represented in equal amounts.

#### 2.1.2. BIO203 Inhibits the Transactivation of PPARs, NF-κB, and AP-1 but Not RXR Transactivation Induced by A2E

**Figure 3:**
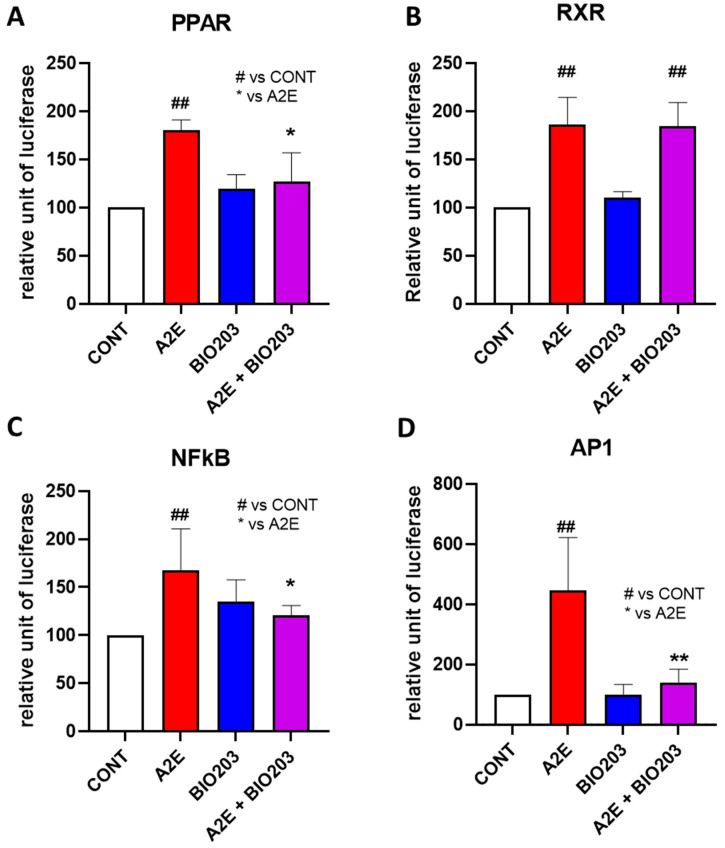
Effect of BIO203 at 20 μM on PPARs, RXR, NF-KB, and AP-1 transactivation induced by A2E at 20 μM in RPE cells in vitro. Bars represent mean ± s.e.m. with n = 3–4 per group. * *p* < 0.05, ## or ** *p* < 0.01, compared to CONT (One-way ANOVA, Dunnett’s post-test).

We have shown previously that norbixin behaves as an inhibitor of A2E-induced PPAR transactivation [5]. In the present study, we tested whether BIO203 at 20 μM had the same effect on these nuclear receptors. Here we report that BIO203 at 20 μM entirely inhibits the PPAR transactivation induced by A2E at 20 μM (Figure 3A). We previously showed that norbixin significantly inhibited A2E-induced transactivation of RXR in porcine RPE cells in vitro [5]. Here, we confirm our previous observation that A2E induces the transactivation of endogenous RXR in porcine RPE cells (Figure 3B). However, BIO203 did not inhibit the transactivation of RXR induced by A2E (Figure 3B), indicating that BIO203 does not share a complete similarity in its mode of action with norbixin. Nevertheless, we showed previously that inhibiting the transactivation of either PPAR or RXR or both resulted in a reduction of inflammation and angiogenesis in our in vitro model [5]. Therefore, we wanted to determine whether BIO203 at 20 μM could also limit inflammation induced by A2E (20 μM) on RPE cells in vitro. Previously, we demonstrated that norbixin inhibits A2E-induced NF-κB and AP1 transactivation, two transcription factors involved in the regulation of inflammation [5]. In the present study, we showed that NF-κb transactivation induced by A2E was significantly downregulated by BIO203 at 20 μM (−69.4%, *p* < 0.05) (Figure 3C) in a similar fashion as norbixin (−73%) [5]. Similarly, A2E-induced AP1 transactivation was also inhibited by BIO203 at 20 μM (Figure 3D). Altogether these observations suggest that BIO203 and norbixin share the same anti-inflammatory effect and similar modulation of PPAR nuclear receptors.

#### 2.1.3. BIO203 Inhibits the Expression of IL-6, IL-8, and VEGF, Induced by A2E

**Figure 4:**
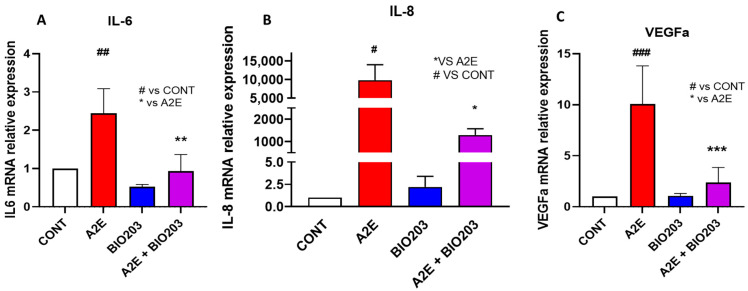
Effect of BIO203 (20 μM) on IL-6 (**A**), IL-8 (**B)**, and VEGF **(C)** expression induced by A2E (20 μM) in RPE cells in vitro. Bars represent mean ± s.e.m. with n = 3–4 per group. # or * *p* < 0.05, ## or ** *p* < 0.01, ### or *** *p* < 0.001, compared to CONT (One-way ANOVA, Dunnett’s post-test).

Transactivation of NF-κb and AP-1 are pivotal in inflammatory responses regulating multiple aspects of innate and adaptative immune functions as well as angiogenesis [9,10]. Here, we aimed to assess the effects of BIO203 at 20 μM on A2E-induced expression of inflammatory cytokines and VEGF, a predominant proangiogenic factor. We have shown previously that norbixin at 20 mM significantly reduced the mRNA expression of IL-6, IL-8, and VEGF [5]. Similarly, BIO203 (20 mM) completely abrogated the mRNA expression of IL-6 to lower levels than baseline without A2E induction (−104.8%, *p* < 0.001) (Figure 4A), IL-8 (−86.8%, *p* < 0.001) (Figure 4B) and VEGF (−84.8%, *p* < 0.001) (Figure 4C) in porcine RPE cells stimulated by 20 μM of A2E. Based on these observations, it could be hypothesized that the BIO203 inhibition of PPAR transactivation induced by A2E might be involved in the inhibition of inflammation and angiogenesis induced by A2E in RPE cells in vitro.

### 2.2. BIO203 Displays Improved Ocular Pharmacokinetics Compared with Norbixin (BIO201) Following Single and Multiple Intraperitoneal Administrations

**Figure 5:**
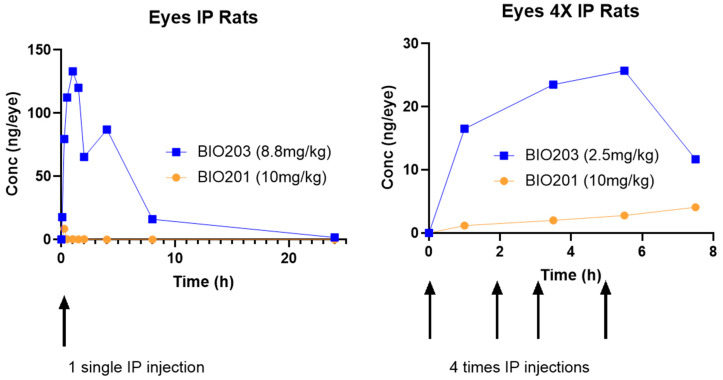
Comparative PK following single or multiple intraperitoneal administrations of BIO203 or norbixin (BIO201). In both experiments, the two eyes from three rats per time point and per group were collected for intraocular concentrations (see material and methods section).

We performed comparative pharmacokinetic studies of BIO203 with norbixin (BIO201) in the plasma and eyes of rats administered intraperitoneally with similar doses of norbixin (10 mg/kg) and BIO203 (8.8 mg/kg). In this experiment, BIO203 displayed increased intraocular AUC and Cmax compared to norbixin (Figure 5A). BIO203 displayed a Cmax value of 133.1 ng/eye at 1 h post-administration, compared to a norbixin Cmax value of less than 8.5 ng/eye at 0.25 h (Figure 5A). In addition, AUC for BIO203 (704.1 ng·h/eye) was approximately 280 times higher than norbixin’s (AUC 2.5 ng·h/eye) (Figure 5A). The remaining intraocular concentration of BIO203 at 24 h was close to 2 ng/mL. In comparison, at this time point, norbixin was no longer detectable in ocular tissues (Figure 5A). We then performed a pharmacokinetic comparison of four intraperitoneal administrations of 10 mg/kg of BIO201 and 2.5 mg/kg of BIO203 in Wistar rats. Despite a higher quantity of norbixin administered, ocular exposure of BIO203 (144.9 ng·h/eye) was approximately 10 times higher than that of BIO201 (16.7 ng·h/eye) (Figure 5B). Altogether, following single and multiple intraperitoneal administrations in rats, BIO203 displays improved ocular pharmacokinetics compared to BIO201. 

### 2.3. BIO203 Is Neuroprotective and Preserves the Visual Function of Albino Rats Exposed to Blue-Light-Induced Photoreceptor Degeneration

**Figure 6:**
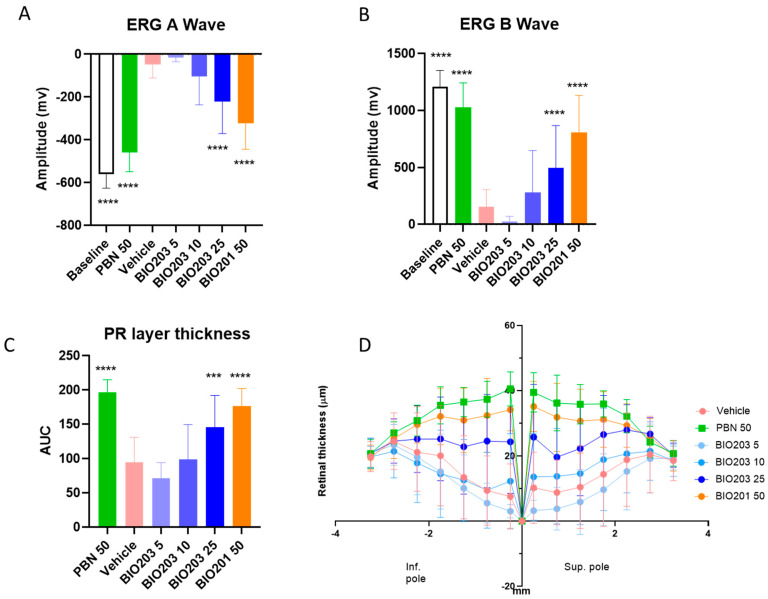
Effect of norbixin and BIO203 on ERG and retinal phototoxicity after BLD in albino rats. (**A**) Photopic A wave and (**B**) Photopic B wave, ERG recorded 7 days after blue-light illumination. (**C**) Graph showing the number of photoreceptor layers measured along the retina, each 200 μm from the optic nerve. (**D**) Histograms showing the area under the curve (AUC) calculated from the photoreceptor layer quantification and used to perform statistical analyses. The doses of PBN (used as a positive control), BIO203, and norbixin are expressed in mg/kg. Bars represent mean ± s.e.m. with n = 10 animals per group (20 eyes analyzed). *** *p* < 0.001, **** *p* < 0.0001 compared to the vehicle group (One-way ANOVA, Dunnett’s post-test).

We previously demonstrated that intraperitoneal injections of norbixin at 50 mg/kg was the lowest dose of BIO201 giving the maximal retinal neuroprotection and optimal preservation of visual functions in albino rats exposed to blue-light illumination [4]. To determine whether systemic administration of BIO203 was neuroprotective and could preserve visual function of photoreceptors in vivo, we used the same model of BLD in albino rats. We performed preliminary experiments comparing doses ranging from 0.125 mg/kg to 2.5 mg/kg of BIO203. The tested doses of BIO203 were chosen according to ocular PK data in rats following the same administration protocol used in the BLD studies presented above (Figure 5B). However, in this preliminary experiment, only a limited neuroprotective effect of BIO203 at 2.5 mg/mL compared to vehicle alone was observed. Therefore, we decided to increase the dose of BIO203 from 5 to 25 mg/kg. Increased doses of BIO203 (5, 10, and 25 mg/kg) were injected intraperitoneally at four-time points: 30 min prior to BLD and 1, 2.5, and 4 h after the beginning of exposure to blue- light. The BIO203 effects on visual function and retinal histology at these three doses were compared to the effect of similar regimen administrations of vehicle and positive controls (PBN at 50 mg/kg and the optimal dose of norbixin (BIO201) at 50 mg/kg). Six hours of blue-light exposure induced severe loss of retinal function in vehicle-dosed rats, as measured seven days after exposure by ERG A-wave (Figure 6A) and ERG B-wave (Figure 6B). As previously demonstrated, treatments with PBN and norbixin protected the visual functions [4]. While there was no significant effect in the group treated with 5 and 10 mg/kg of BIO203, four consecutive intraperitoneal administrations of BIO203 at 25 mg/kg provided a protective effect on ERG A-wave and ERG B wave (*p* < 0.0001). This protective effect was like those observed in PBN and norbixin-treated animals (Figure 6A,B). We then evaluated the neuroprotective effect of intraperitoneal injections of BIO203 at 5, 10, and 25 mg/kg. In agreement with the effects observed on visual function, treatment with PBN, norbixin (BIO201) at 50 mg/kg, and BIO203 at 25 mg/kg partially preserved the retinal structure of rats subjected to 6 h of blue-light exposure (*p* < 0.0001) (Figure 6C,D). By contrast, we did not observe neuroprotection following treatment with lower doses of BIO203 at 5 and 10 mg/kg and with vehicle (Figure 6C,D). Altogether we show here that intraperitoneal injections of BIO203 are neuroprotective and partially preserve visual function in vivo in a model of blue-light damage in rats.

### 2.4. Effect of 6 Months BIO203 Oral Treatment by Complementation in 11–12-Month-Old Abca4^−/−^ Rdh8^−/−^ Mice

**Figure 7:**
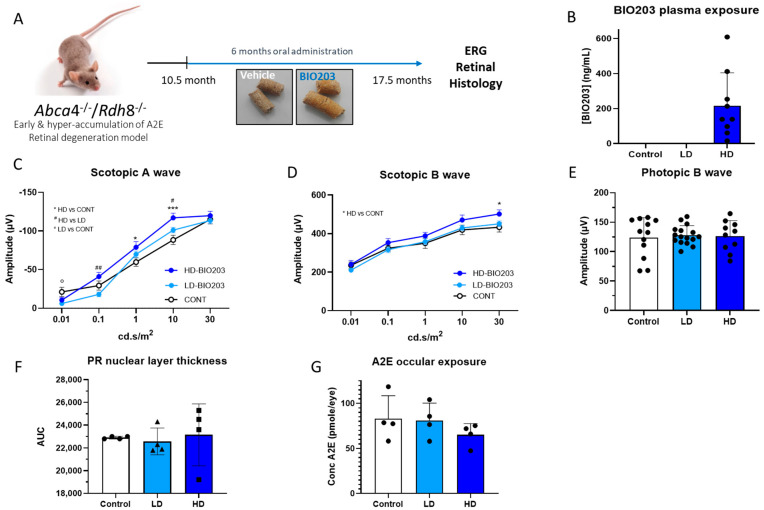
Effects of BIO203 treatment by oral complementation in *Abca4^−/−^ Rdh8^−/−^* mice. (**A**) Schematic representation of the 6-month treatment with pellets containing BIO203. (**B**) Plasma exposure of BIO203 following treatment with LD- (0.05 g/Kg) and HD- (0.5 g/kg) BIO203 containing pellets in *Abca4^−/−^ Rdh8^−/−^* mice (n = 9 per group) (**C**) Scotopic A wave ERG recorded after 6 months of oral treatment with LD- and HD- BIO203 containing pellets in *Abca4^−/−^ Rdh8^−/−^* mice compared to mice fed with control chow (CONT) (n = 5–9). (**D**) Scotopic B wave (n = 5–9). (**E**) Photopic B wave (n = 5–8). (**F**) Quantification of photoreceptor nuclear layers along the superior and inferior poles of the retina, each measured every 200 μm apart from the optic nerve. (**G**) A2E quantification in eyes (n = 4 per group) from 17–18-month-old *Abca4^−/−^ Rdh8^−/−^* mice fed with normal chow or with LD- or HD-BIO203-containing pellets. N.T.: not tested, N.D. Not detected. Bars represent mean ± s.e.m. * *p* < 0.05, *** *p* < 0.001, HD compared to vehicle, ° *p* < 0.05, LD compared to vehicle, ^#^ *p* < 0.05 and ^##^ *p* < 0.01 HD compared to LD (One-way ANOVA, Dunnett’s post-test).

Eleven to twelve-month-old *Abca4*^−/−^ *Rdh8*^−/−^ mice were fed either with normal pellets (control) or with pellets containing BIO203 (LD-BIO203 [50 μg/g] and high dose HD-BIO203 [500 μg/g]) for 6 months (Figure 7A). Firstly, we determined whether BIO203 could be detected in the eyes and plasma of mice fed with BIO203 (Figure 7B). BIO203 was not detected in the eyes of mice fed during 6 months with LD or HD -BIO203-containing pellets; however, in the plasma, BIO203 was detected in the group treated with HD-BIO203 but not with LD-BIO203 (Figure 7B). After 6 months of treatment with HD-BIO203 pellets, the scotopic A wave ERG (at flash intensities: of 1 and 10 cd.s/m^2^) was significantly superior when compared with the ERG of animals fed with control pellets (Figure 7C; *p* < 0.05). Similarly, 6 months of complementation with HD-BIO203 reduced the loss of scotopic B wave ERG (at flash intensity: 30 cd.s/m^2^) when compared with the intensity of scotopic B wave of animals treated with control pellets (Figure 7D; *p* < 0.05). However, no significant difference in photopic B wave ERG was observed between the group of mice treated with HD-BIO203 compared to the group of mice fed with control pellets (Figure 7E). No effect on the visual function was observed in the group of mice treated with LD-BIO203 (Figure 7C–E). The lack of effect of LD-BIO203 is in agreement with the absence of BIO203 plasmatic exposure in this group of mice and indicates a dose-response effect. No difference in the thickness of the photoreceptor nuclear layer was noted between eyes of 17-month-old mice treated with HD-BIO203 compared to eyes of mice fed with LD-BIO203 or control pellets for the previous 6 months (Figure 7F). In addition, we previously reported that norbixin reduced A2E accumulation in vitro [4] and in vivo in 17-month-old mice treated the previous 6 months by norbixin [7]. Here, we measured the amount of A2E in the retina of mice treated with control or with LD- or HD-BIO203 for 6 months. The amounts of ocular A2E did not differ significantly between mice treated with control pellets and with LD- and HD-BIO203-containing pellets during the 6 months (Figure 7G). However, we observed a trend towards a reduction of A2E concentration in the retinas of animals treated with HD-BIO203 compared to mice that were fed with control pellets (Figure 7G), but this difference did not reach significance (−21.7%; *p* = 0.407).

## 3. Discussion

Age-related macular degeneration (AMD) is the most frequent cause of severe visual function loss and blindness in developed countries among individuals aged 60 and older [1]. AMD is still a major unmet medical need, and the development of new therapeutic strategies to fight this blinding disease are still required. The major risk factors remain aging [11,12] along with smoking [13]. In addition, oxidative stress, angiogenesis, and inflammation have been defined as critical factors for retinal degeneration leading to AMD pathogenesis [14,15,16]. *N*-retinylidene-*N*-retinylethanolamine (A2E) accumulation in the retina and retinal pigmented epithelium (RPE) cells has been associated with the initial stage of the disease [17,18,19,20]. An important role for nuclear receptors in the pathogenesis of AMD has been strongly suggested [21,22,23,24]. We have previously shown that A2E activates PPAR-α, PPAR-β/δ, PPAR-γ, and RXR, and we proposed that this could lead to the cascade of inflammation, angiogenesis, and retinal degeneration observed during AMD [5]. Indeed, we have shown that 9′-*cis*-norbixin, a 6,6′-di-*apo*-carotenoid extracted from annatto (*Bixa orellana*), behaves as an antagonist of PPARs and RXR transactivation induced by A2E and counteracts its proinflammatory and proangiogenic effects [5]. For instance, in vitro, norbixin leads to the photoprotection of primary porcine RPE cells from A2E and blue-light illumination in rats [4] and mice [7]. Moreover, norbixin regulates inflammation, as demonstrated by the inhibitory effect of BIO201 on A2E-induced expression of IL-6, IL-8, transactivation of NF-κB, and AP-1 [5]. In addition, our previous observations demonstrated that norbixin impairs VEGF expression induced by A2E [5]. We also demonstrated the neuroprotective effects and the preservation of visual function, especially of rod photoreceptors by BIO201 in various in vivo animal models of AMD [4,7] and as previously demonstrated for retinal carotenoids such as lutein, norbixin reduced the accumulation of A2E [4,7,25]. These promising observations suggested that BIO201 could be developed as a new treatment for the initial stages of AMD [2]. However, we noted that ocular accumulation of BIO201 administered systematically was limited [7] and that its poor stability was not compatible with its industrial development as an effective oral drug to treat AMD.

We developed by hemisynthesis a second generation of molecules. Starting from bixin, we reacted the free carboxylic group with a set of primary or secondary amines. We found that some of the resulting amides had a better pharmacokinetic profile and a better tropism for the eye than those of norbixin (patent Dinan et al., 2019; US 17/788,534).

In the present study, we describe the properties of BIO203, one of these norbixin conjugates. Here, we report that BIO203 has improved stability and ocular pharmacokinetics, with both C_max_ and AUC increased by more than 10- and 200-fold compared to BIO201, respectively. The exact mechanisms explaining these improved properties remain unknown at present but could be due to a better ocular uptake of BIO203 originating from the plasma. It is perhaps possible that the addition of the amine group modifies the polarity of BIO203, which becomes more lipophilic. Further experiments to decipher the mechanisms at play are currently underway. We also observed an equivalent or slightly improved photoprotective efficacy of BIO203 compared to norbixin in the phototoxicity test in RPE cells challenged with blue-light and A2E in vitro at low concentrations (Figure 2). Interestingly, in vitro, the effect on the nuclear receptors PPARs and RXR of norbixin and its conjugate BIO203 were similar but not identical. Indeed, both norbixin and BIO203 inhibited equally PPARs transactivation induced by A2E. We show here that, contrary to norbixin, BIO203 does not inhibit RXR transactivation. Nevertheless, and probably due to the permissive nature of PPARs that form heterodimers with RXR [26,27,28,29], disrupting the binding of A2E with PPARs or RXR alone is sufficient to inhibit the inflammatory and angiogenic effects of A2E [5]. In agreement with this observation, BIO203, which only impairs PPARs transactivation but not RXR transactivation induced by A2E, reproduces all the anti-inflammatory and anti-angiogenic properties observed with norbixin. Indeed, we show here that BIO203 inhibits NF-κB and AP-1 transactivation induced by A2E. Accordingly, BIO203 also reduced IL-6, IL-8, and VEGF expression induced by A2E in primary porcine RPE. In addition, BIO203 slightly inhibited IL-6 expression on its own, but this tendency was not statistically significant. In vivo, repeated intraperitoneal injections of BIO203 protected visual function in albino rats exposed to blue-light exposure as previously observed with norbixin [4]. Indeed, BIO203 had a protective effect on scotopic and photopic ERG amplitudes similar to norbixin. Moreover, BIO203 as norbixin was able to reduce retinal degeneration in this acute blue-light exposure model in rats.

We then evaluated the effects of long-term (6-months) oral treatment by complementation of two doses of BIO203 in ABCA4^−/−^ RDH8^−/−^ mice accumulating A2E during aging. Here we showed that the highest dose of BIO203 partly but significantly preserved scotopic A wave and B wave amplitude. By contrast, BIO203 did not limit retinal degeneration in ABCA4^−/−^ RDH8^−/−^ mice. Since BIO203 demonstrated neuroprotective properties in the albino rat model, we hypothesize that the ABCA4^−/−^ RDH8^−/−^ mice used in the present study were probably too old (12–17 months) to observe an effect. Indeed, we previously demonstrated that the maximum retinal degeneration in this animal model occurs in animals aged between 9 and 15 months [7]. Accordingly, BIO203 did not show an effect on photopic vision and A2E accumulation in mice. However, we observed a non-significant reduction (−21.7%; *p* = 0.407) of A2E levels in the eyes of mice treated with HD-BIO203. Future experiments with higher numbers of animals will be required to confirm this trend. Importantly, there was a clear dose-response effect in these experiments performed in mice. Indeed, we could not detect BIO203 in the plasma of mice treated with low LD-BIO203-containing pellets, in which we also did not observe an effect of BIO203 in vivo. In contrast, preservation of visual function was observed in mice treated with HD-BIO203 and in which BIO203 could be detected in the plasma. Therefore, it can be hypothesized that increasing the dose of BIO203 further would allow to reproduce all the effects on retinal and visual functions in mice previously observed in mice treated with norbixin. Although the effects of HD-BIO203 were limited to the preservation of scotopic ERG, it is important to remind that protection of scotopic vision is crucial for the cure of the early stages of AMD. Indeed, the first clinical sign of intermediate AMD is the loss of “night vision” mediated mainly by rod photoreceptors whose activity is measured by scotopic ERG [2,30,31]. We also observed a dose-response effect of BIO203 in the rat BLD-model as shown in our preliminary experiments ranging from 0.125 mg/kg to 2.5 mg/kg and the experiments presented here with doses ranging from 5 to 25 mg/kg. The efficacy dose of BIO203 and norbixin in the rat model of BLD were similar (25 and 50 mg/kg, respectively) despite their different pharmacokinetics. The reason for this apparent absence of correlation between improved ocular uptake into the eye and an expected reduced efficacious dose of BIO203 is not understood at the moment and will be the subject of further pharmacological and toxicological studies that are required before evaluating the safety and efficacy of BIO203 in clinical studies in humans.

In conclusion, norbixin (BIO201) efficacy in an in vitro model of RPE dysfunction and in vivo models of AMD using several strains of mice (WT and genetically modified (ABCA4^−/−^RDH8^−/−^) double knock-out mice) and WT rats have been presented in our three previous articles: [4,5,7]. In the present study, we have demonstrated that BIO203, which displays improved ocular PK characteristics and stability when compared to norbixin, reproduces the effects previously observed with norbixin. These include photoprotection in vitro, inhibition of transactivation of NF-κB and AP-1, and the expression of VEGF, IL-8, and IL-6, involved respectively in angiogenesis, and inflammation, which are critical for AMD pathogenesis. All these effects may be at least partially linked to their inhibition of PPAR transactivation induced by A2E. We also report that in vivo, BIO203 administered systemically is neuroprotective and has limited visual function loss, as norbixin did, in two models of retinal degeneration. We previously reviewed the potential clinical interest of carotenoids in general for the treatment over long periods of time (years) in patients diagnosed with the intermediate form of AMD [2]. Our results of visual function protection in ABCA4^−/−^RDH8^−/−^ mice fed with pellets containing HD-BIO203 for 6 months suggest that this new compound could be well suited for the treatment of the initial stages of the disease and particularly for patients with early and intermediate AMD. BIO203, with improved eye exposure and stability compared to norbixin, may be an even better drug candidate for the treatment of AMD. In consequence, Biophytis is actively engaged in the clinical development of BIO203 in this indication.

## 4. Material and Methods

### 4.1. Ethics Statement

All procedures were carried out according to the guidelines on the ethical use of animals from the European Community Council Directive (86/609/EEC) and were approved by the French Ministry of Agriculture (OGM agreement 6193) and by the Committee on the Ethics of Animal Experiments of the French Ministry of Research. All efforts were made to minimize suffering.

### 4.2. Reagents/Chemicals/HPLC Equipment

All usual chemicals and primers were from Sigma (St. Louis, MO, USA). Reagents for cell culture, transfection, and quantitative RT-PCR were from Thermo Fisher Scientific (Waltham, MA, USA). RNA extraction NucleoSpin^®^ RNA kit was from Macherey Nagel (Düren, Germany). Cignal Pathway Reporter Assay Kits were from QIAGEN (Frederick, MD, USA). The Dual-Luciferase Reporter Assay System was purchased from Promega (Madison, WI, USA).

All HPLC analyses were performed on an Agilent 1260 equipped with a DAD and a triple quadrupole mass spectrometer (API6420, Applied Biosystems, Les Ulis, France). Analytical conditions are indicated for the different uses in the appropriate sections.

### 4.3. Preparation of BIO201 and BIO203

**Figure 8:**
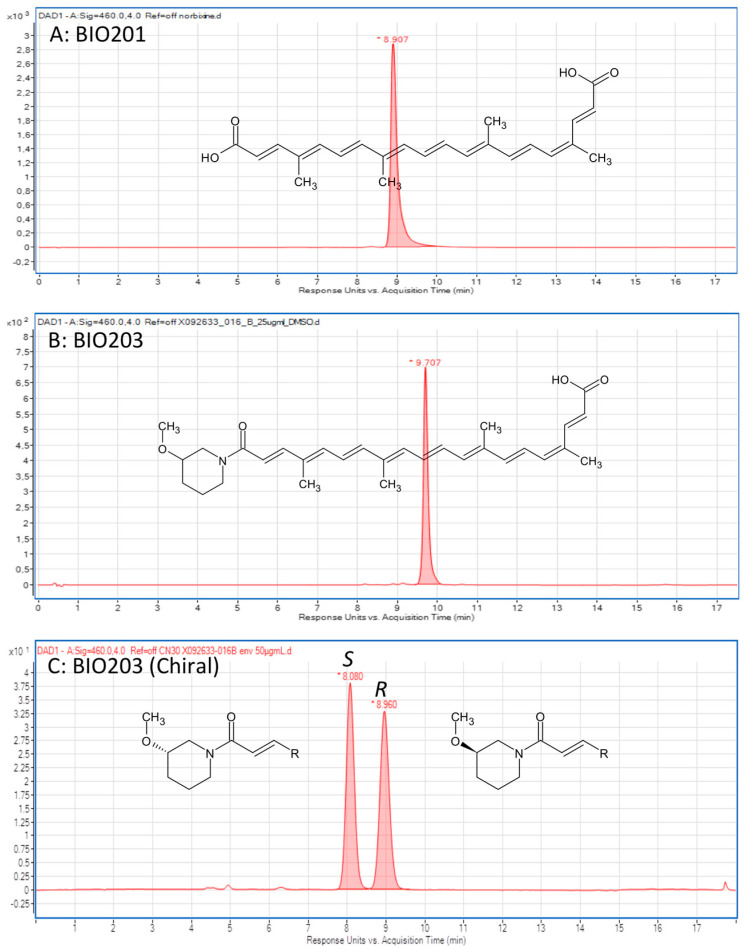
Structures and HPLC analysis of the compounds tested in the study. (**A**): Norbixin (BIO201) and (**B**) BIO203 structures and HPLC analysis showing their 97% and 98% purity, respectively. HPLC analytical conditions: Column Fortis C18 5 µm (2.1*50 mm), flowrate 300 µL/min, gradient from 35% to 100% of acetonitrile (+0.1% formic acid) in water (+0.1% formic acid) in 10 min, then rinsing and return to initial conditions, total run time 18 min, detection at 460 nm. (**C**): HPLC separation of the two *R* and *S* enantiomers of BIO203 on a Chiralpak IH-3 column (3 µm, 4.6 mm × 150 mm), flowrate 1 mL/min, isocratic 60% of acetonitrile (+0.1% formic acid) in water (+0.1% formic acid) for 12 min, then rinsing with 95% acetonitrile (3 min) and return to 60%.

BIO201 (9′-*cis*-Norbixin) (Figure 8A) was prepared from 9′-*cis*-Bixin (AICABIX P, purity 92%) purchased from Aica-Color (Cusco, Peru) upon alkaline hydrolysis as previously described [4,32]. The obtained product showed an HPLC purity of 97% (Figure 8A), and its 9′-*cis* structure was confirmed by NMR as previously described [33] (Table 1). BIO201 was stored as a red powder at −80 °C, and fresh solutions were prepared in DMSO.

BIO203 (Figure 8B) was synthesized in two steps:

Peptide coupling: Away from light and under a nitrogen atmosphere, bixin (7 g, 17.8 mmol) was suspended in anhydrous DMF (56 mL). Triethylamine (7.4 mL, 53.4 mmol) and carbonyldiimidazole (5.77 g, 35.6 mmol) were then added, and the mixture was stirred at room temperature for 2 h. 3-methoxypiperidine hydrochloride (8.10 g, 54.4 mmol) was added, and the mixture was stirred overnight at room temperature. Then 200 mL of water was added portion-wise, and the mixture was stirred for 1 h at room temperature after the end of the addition. The mixture was filtered onto a glass frit, and the purple paste was washed with water (3 × 40 mL), 1N HCl aqueous solution (3 × 40 mL), and di-isopropyl ether (3 × 40 mL). The resulting paste was dried on the glass frit and was used without further purification (kept at −20 °C).

Hydrolysis step: Away from light, the paste (ester) (23.1 g, max 17.8 mmol) was dissolved in THF/MeOH (175 mL/90 mL) solution. 1N NaOH aqueous solution (90 mL) was added, and the mixture was stirred overnight at room temperature. 1N HCl aqueous solution (90 mL) was slowly added, and the mixture was stirred for 20 min at room temperature. The mixture was filtered through a glass frit, and the residue was washed with water (120 mL, then 2 × 65 mL). The red paste was solubilized in water/acetonitrile solution and then lyophilized to result in BIO203 as a red powder (6.96 g, r = 82%). The 9′-*cis* structure and purity of BIO203 at 98% were assessed through HPLC (Figure 8B) and NMR analysis (Table 1). Given the racemic structure of the 3-methoxypiperidine used, the resulting BIO203 is also a racemate, and the *R* and *S* enantiomers can be resolved by HPLC on a chiral column (Figure 8C). The assignment of R or S structures was unambiguously established by the synthesis of pure enantiomers by using the same protocol with pure (R)- or (S)-3-methoxypiperidine.

### 4.4. Evaluation of Norbixin (BIO201) and BIO203 Powder Stability at Room Temperature, 4 °C, and −20 °C

The potential breakdown of BIO203 compared to BIO201 was assessed every three months in samples stored for up to 18 months at room temperature, 4 °C, and −20 °C. On the day of analysis, weighted samples of BIO203 and norbixin powder were solubilized in DMSO at 10 mg/mL final concentration. DMSO solutions were further diluted 400-fold before performing LC-MS/MS analysis which was performed on an LC 1260 System coupled with Mass Spectrometer QQQ-6420 with DAD (Supplier Agilent Technologies). BIO201 and BIO203 were eluted from a reverse-phase column (2.1 mm × 50 mm; 5 μm particles; Fortis C18) with the following gradient of acetonitrile in water (both containing 0.1% formic acid): 10 to 100% in 10 min), (flow-rate: 0.3 mL/min), (UV detection A: 460 nm, B: 254 nm, DAD: 210–500 nm), (MS scan+: 50 to 800 Frag 120 CAV 5, MS scan-: 50 to 800 Frag 120 CAV 5). The AUC value was used to determine the stability of each compound.

### 4.5. Synthesis of A2E

A2E (*N*-retinylidene-*N*-retinylethanolamine) was synthesized by Orga-link (Magny-Les- Hameaux, France) as described before [4].

### 4.6. RPE Phototoxicity and Cytokines Expression In Vitro

RPE cells were obtained from pig eyes, as previously described [4]. RPE cells were seeded on a 96-well plate at a density of 1.5 × 10^5^ cells/cm^2^ in DMEM 2% FCS. BIO201, BIO203, or its pure enantiomers were added in increasing concentrations to the culture medium 48 h before illumination. A2E was added to the medium at a final concentration of 30 μM, and 19 h later, blue-light illumination was performed for 50 min using a 96 blue-led device (Durand, St Clair de la Tour, France) emitting at 470 nm (1440 mcd, 8.6 mA). Just before illumination, the culture medium was replaced by a modified DMEM without any photosensitizer and with 2% FCS. Twenty-four hours after blue-light irradiation, all cell nuclei were stained with Hoechst 33342, and nuclei of dead cells with ethidium homodimer 2, fixed with paraformaldehyde (4% in PBS, 10 min) and 9 pictures per well were captured using a fluorescence microscope (Nikon TiE) equipped with a CoolSNAP HQ2 camera and driven by Metamorph Premier On-Line program. Quantification of live cells was performed using Metamorph Premier Off-Line and a homemade program by subtraction of dead cells from all cells. For cytokines mRNA analysis, RPE cells were seeded in 24-well plates and left untreated or treated with A2E or BIO203 alone or A2E plus BIO203. After 48 h, cells were lysed using the lysis buffer from the NucleoSpin^®^ RNA kit, and the samples were stored at −80 °C.

### 4.7. PPAR, RXR, AP-1, and NF-κB Transactivation Assays

Transactivation assays were performed as previously described [4]. Briefly, RPE cells were plated in DMEM 2% FCS at a density of 6 × 10^4^ cells/cm^2^. The next day transfections using the Cignal Reporter Assay Kits for PPAR, RXR, AP-1, and NF-κB were performed with Lipofectamine and Plus Reagent in serum-free medium. Three hours after the transfection, A2E and/or BIO203, both at 20 μM, were added to the culture medium for 24 h. Luciferase activity was measured using the Dual-Luciferase Reporter Assay System and with a luminometer (Infinite M1000 from Tecan, Mannedorf, Switzerland). At least 3 independent transfections were performed in triplicate for each condition.

### 4.8. Quantitative RT-PCR

Total RNA was extracted using the NucleoSpin^®^ RNA kit. Reverse Transcription of 500 ng of RNA was performed using the SuperScript III Reverse Transcriptase. Five ng of cDNA were amplified using the SYBR GREEN real-time PCR method. PCR primers for target genes and housekeeping gene GAPDH were designed using Primer3Plus Bioinformatic software (Table 2). RT-PCR conditions have been described previously [4]. All experimental conditions were processed in triplicate, and each experiment was done at least 3 times.

### 4.9. Acute Ocular PK Studies of Norbixin (BIO201) and BIO203 in Rats Following a Single IP Administration

The compounds were administered to 8–9 months-old Male Sprague-Dawley rats (n = 3 per group and per collection time) by the intraperitoneal route in vehicle (DMSO/NaCl/NaOH for BIO203 and PBS/Tween 80 (95/5) for norbixin) with an administration volume of 10 mL/kg. The solutions of BIO203 and norbixin were centrifuged, and the clear supernatants were administered to rats. The final injected concentrations were 10 mg/kg and 8.8 mg/kg for norbixin and BIO203, respectively. Doses administered differ because BIO201 and BIO203 solubility are not equivalent in their respective vehicles, and the dose of BIO203 was reduced to avoid toxicity of DMSO present in the vehicle, but the same volumes were injected into animals. Eyes were collected post-administration according to the following timing: 0, 0.08 h, 0.25 h, 0.5 h, 1 h, 1.5 h, 2 h, 4 h, 8 h, and 24 h. After euthanasia, the two eyes of each rat were collected in a Precellys tube, frozen, and stored at −80 °C.

### 4.10. Ocular PK Studies of BIO201 and BIO203 in Rats Following Four Repeated IP Administrations

The compounds were administered to 8–9 months old Male Sprague-Dawley rats (n = 3 per group and per collection time) by four successive intraperitoneal administrations in vehicle (DMSO/NaCl/NaOH for BIO203 and PBS/Tween 80 (95/5) for BIO201). The solutions of BIO203 and BIO201 were centrifuged, and the clear supernatants were administered to rats. Administered volume was 10 mL/kg, and the final injected concentrations were 10 mg/kg and 2.5 mg/kg for BIO201 and BIO203, respectively. Again, doses administered differ because BIO201 and BIO203 solubility are not equivalent in their respective vehicles. The dose of BIO203 was reduced to avoid toxicity of DMSO present in the vehicle, but the same volumes were injected into animals. The eyes were collected 1 h after administrations n°1 to 4 according to the following timing: 1 h, 3.5 h, 5.5 h, and 7.5 h. After euthanasia, the two eyes of each rat were collected in a Precellys tube, frozen, and stored at −80 °C.

### 4.11. Electroretinogram (ERG) in Rats and Mice

The ERG in rats was performed using the electrophysiological system RETI-animal^®^ from Roland Consult. ERG was recorded on both eyes from overnight dark-adapted animals. Rats were anesthetized by an intramuscular injection of a mix of Rompun^®^ (xylazine)/Imalgene^®^ (ketamine) before ERG measurement. Ten to fifteen minutes before measurement, one drop of Mydriaticum^®^ (0.5% tropicamide) was instilled for pupillary dilatation. The A-wave and B-wave implicit times (ms) and amplitudes (µV) were measured for each ERG. The B-wave amplitude (value and %) was given as informative data. ERG parameters: Ganzfeld Q450C, Color: white maximum, Maximum intensity: 3 cd/m^2^ (0 dB); Duration 0.24 ms; number of flashes: 1, Filter: 50 Hz, Impedance threshold: 50 kΩ. For scotopic ERGs, the A-wave amplitudes were measured from the baseline to the peak of the negative potential, whereas B-wave amplitudes were measured from the trough of the A-wave to the peak of the positive potential. The recordings obtained from these analyses were tabulated and normalized (%) for each eye. Group means, standard deviation, and median values were calculated, considering each eye from one animal as a separate value. ERG recordings in mice were performed as previously described [7].

### 4.12. Histology and Measurement of the Retina Outer Nuclear Layer (ONL) Thickness in Rats and Mice

At the end of the measurement period on Day 7, rats were euthanized by an intraperitoneal injection of pentobarbital. Immediately after euthanasia, both eyeballs were sampled, fixed in Davidson’s solution, and processed for histology. Sections (5 to 7 µm thick) were performed along the vertical meridian and stained with Haematoxylin/Eosin stain. The vertical meridian included the optic nerve. ONL Thickness was first measured at 250 µm from the optic nerve and then every 500 µm (seven points in total) to the peripheral retina in each part of the retina (superior and inferior) using a standard microscope (Leica) on live (pictures were not saved). The thickness of the outer nuclear layer was measured on each point. The ONL areas were calculated by integrating the area under the curve of the retinal thickness from 3.25 mm superior and 3.25 mm inferior to the ON. Histology and photoreceptor counting in mice were performed as previously described [7].

### 4.13. Blue-Light Damage (BLD) Study in Rats

Sixty-four animals were selected based on good health and on scotopic electroretinogram performed before induction when the A-wave amplitude of both eyes was in the range of the global mean (all animals) of A-wave amplitude ± 1.96 SD. Animals were then randomized into the study groups using a macro function in Excel^®^ software based on the A-wave amplitude of both eyes (mean value). On Day 0, BIO203, negative controls, and comparator (N-tert-Butyl-a-phenylnitrone (PBN)) were intraperitoneally injected using a 25 G needle in rats that have been dark-adapted for 36 h. Individually housed rats were then exposed for 6 h (±5 min) to a continuous blue, fluorescent light (400–540 nm) in clear plastic cages. After exposure and administration, the rats were placed in a dark room for 24 h (±1 h) and then were returned to rearing cyclic light conditions.

### 4.14. BIO203-Containing Pellets

A custom rodent diet was formulated and irradiated (25 kGy) by Envigo RMS SARL (Gannat, France). Low dose (LD)-BIO203 (50 μg/g) and high dose (HD)-BIO203 (500 μg/g) were incorporated into Teklad Custom Research Diet 2016© pellets. The pellets were stored at −20 °C until use and were administered ad libitum as the standard Teklad Global Rodent Diet 2016© used for the control group. The concentration of BIO203 in the customized pellets at the end of each batch was determined by HPLC MS/MS. The mean concentration was 46 μg/g ± 3.5 μg/g of pellet for the LD and 464 μg/g ± 24.3 μg/g of pellet for the HD. We calculated that in the LD group, male mice weighing 31.63 g after 2 months of complementation consumed 4.16 g of pellets, and in the HD group, male mice weighing 34.11 g after 2 months of complementation consumed 4.11 g of pellets every day, which correspond to a daily dose of 6.04 ± 0.46 mg/kg for the LD-BIO203 pellets and 55.91 ± 2.93 mg/kg for the HD-BIO203 pellets.

### 4.15. In Vivo Oral BIO203 Treatment by Complementation

To test the preventive/curative action of oral administration of BIO203 against retinal neurodegeneration, a total of 27 Pigmented *Abca4^−/−^ Rdh8^−/−^* mice carrying the Rpe65-Leu450 mutation and the *rd8* mutation in the *Crb1* gene from Case Western Reserve University [34] of eleven to twelve months of age were used. Three groups of 9 males received control chow (Teklad Custom Research Diet 2016© pellets) or chow containing LD-BIO203 or HD-BIO203 orally for 6 months. After 6 months of complementation, ERG was measured in both eyes. In the control group, two mice died during the period of complementation and one mouse during ERG measurement. In the HD-BIO203 group, one mouse died before the end of complementation, and 3 mice died just after anesthesia. All the mice that received LD-BIO203 were alive until the end of the complementation. One of them died during the photopic ERG measurement. Blood was collected by submandibular puncture in all mice before being euthanized. In each group, half of the eyes were removed for A2E and BIO203 measurements, and half of the eyes were used for histological analyses and photoreceptor layer quantification.

### 4.16. Determination of Norbixin (BIO201) and BIO203 Concentrations in Mice Plasma and Eye Samples

Analysis was performed on an LC 1260 System coupled with Mass Spectrometer QQQ-6420 with DAD (Agilent Technologies). Norbixin and BIO203 were eluted from a reverse-phase column (2.1 × 50 mm; 3 μm particles; Ace Excel C18) with the following gradient of acetonitrile in water (both containing 0.1% formic acid): 55 to 100% in 5 min), (flowrate: 0.3 mL/min). BIO203 and its isomers were monitored by a mass spectrometer, in positive MRM mode, with the following transitions, respectively, 381.1 -> 144.9 and 478.1 -> 362.9. For quantification of BIO201 and BIO203, calibration curves were prepared under the same conditions as the sample matrix, with various amounts of BIO201 or BIO203 (25 to 10,000 ng/mL in plasma and 5 to 500 ng/mL for eyes).

For determination of norbixin or BIO203 concentrations in plasma, aliquots of plasma samples (25 μL) and internal standard (retinoic acid at 10 µg/mL) were distributed in a 96-well microtiter plate and precipitated with methanol (100 μL). After 10 min mixing, the microtiter plate was frozen at −20 °C for 30 min, thawed, and then centrifuged. The hydro-alcoholic phase was removed from each well and transferred into another microtiter plate for LC-MS/MS analysis. Under these conditions, with 5 μL injections, the limit of quantification (LOQ) was 25 ng/mL (0.33 pmol for BIO201 and 0.26 pmol for BIO203).

For determination of concentrations of BIO201 or BIO203 in ocular tissues, each eye was homogenized in CHCl_3_/MeOH (1:1, *v*/*v*) (0.5 mL) with a homogenizer (Precellys-24) during 2 cycles (30 s) at 6500 rpm. The internal standard (retinoic acid at 10 µg/mL) was added, and the organic layer was extracted. The homogenate was then extracted two times with CHCl_3_/CH_2_Cl_2_ (0.5 mL). The combined organic extracts were dried in vacuo without heating (EZ2, Genevac Ltd., Ipswich, UK). Then they were dissolved in 100 μL DMSO/MeOH (1:1, *v*/*v*) and transferred to microtiter plates for LC-MS/MS analysis.

### 4.17. A2E Measurement in Mice Eyes

A2E quantification in eyes was performed using the HPLC-MS/MS method, which has been described previously [7].

### 4.18. Statistical Analyses

Statistical significance was determined by applying an analysis of variance (one-way ANOVA for one parameter) or a Kruskal–Walli’s test to assess differences among groups (AUC (ONL)). After a significant ANOVA, comparisons between groups were made with a Dunnett’s, Dunn’s, or Tukey’s test according to the homogeneity of variance. A second analysis with a two-way ANOVA for two parameters (ONL thickness and optic nerve distance) was performed to assess differences among groups. After a significant ANOVA, comparisons between groups were made with Tukey’s test. The significant threshold was fixed at 0.05, i.e., the *p*-value had to be lower than 0.05 to be significant. Tests were performed using Prism 7 (GraphPad Software, La Jolla, CA, USA).

## Figures and Tables

**Table 1:**
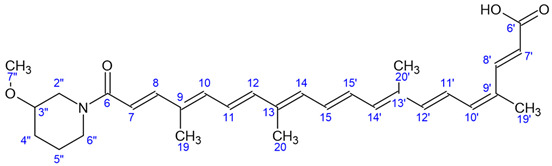
Comparative identification by NMR of protons and carbons in (norbixin) BIO201 and BIO203.

Number H/C	^1^H		^13^C	
	BIO201	BIO203	BIO201	BIO203
6	-	-	168.17	165.8
7	5.89	6.55	119.02	117.3
8	7.36	7.29	149.76	146.9
9	-	-	134.46	135.2
10	6.61	6.52	140.04	138.2
11	6.78	6.77	125.82	125.8
12	6.63	6.58	142.22	141.4/141.3
13	-	-	136.72	137.7
14	6.45	6.44	135.24	135.0/135.4
15	6.82	6.82	132.05	131.9/132.0
15′	6.82	6.82	132.05	131.9/132.0
14′	6.45	6.44	135.24	135.0/135.4
13′	-	-	138.14	137.5
12′	6.53	6.53	141.15	141.4/141.3
11′	6.98	6.98	124.26	124.0
10′	6.47	6.47	138.3	138.5
9′	-	-	132.48	132.3
8′	7.96	7.97	140.85	140.9
7′	5.92	5.91	116.9	119.0
6′	-	-	168.17	168.0
19	2.00	2.00	12.13	13.0/12.9/12.8
20	2.02	2.04	12.33	13.0/12.9/12.8
20′	2.02	2.01	12.44	13.0/12.9/12.8
19′	1.99	1.98	19.89	20.3
2″a 2″b	-	4.00; 3.75 3.50; 3.27	-	nd
3″	-	3.25	-	75.8; 75.5
4″a 4″b	-	1.94 1.58	-	nd
5″a 5″b	-	1.74 1.42	-	nd
6″a 6″b	-	3.73; 3.58 3.50; 3.44	-	nd
7″	-	3.32	-	56.2

Note: NMR spectra were acquired on a 500.45 MHz Bruker Avance II spectrometer equipped with a 5 mm ^1^H/^13^C dual cryo-probehead. Lyophilized powders were dissolved in 0.55 mL of acetone (d6) 99.8%. NMR experiments were conducted as previously described [10] to obtain 1D ^1^H, 2D ^1^H-^1^H TOCSY and NOESY, 2D ^1^H-^13^C HSQC, and HMBC spectra. In addition, 1D ^13^C{^1^H} UDEFT experiments were performed using the pulse sequences *udeft* from the Bruker library to improve the acquisition time and quality of 1D ^13^C{^1^H}. Standard parameters were set, with 2048 scans and 8 dummy scans, and a recycling delay of 3 s. The spectral window used was 236.5 ppm.

**Table 2:** Probes used for mRNA quantification by RT-QPCR.

Gene	Sequence	
GAPDH	F	GCTGCTTTTAACTCTGGCAA
	R	CCACAACATACGTAGCACCA
IL-6	F	CGGATGCTTCCAATCTGGGT
	R	CACAGCCTCGACATTTCCCT
IL-8	F	GGCAGTTTTCCTGCTTTCT
	R	CAGTGGGGTCCACTCTCAAT
VEGF A	F	GTCTGGAGTGTGTGCCCA
	R	GTGCTGTAGGAAGCTCATC

## Data Availability

The data presented in this study are available on request from the corresponding author. The data are not publicly available due to the fact they are the private property of Biophytis.
